# Intensified anti-TNF treatment downregulates the phenotype in ulcerative colitis: a 13-year prospective follow-up study

**DOI:** 10.3389/fgstr.2023.1304944

**Published:** 2024-01-24

**Authors:** Jon Florholmen, Rasmus Goll, Kay-Martin Johnsen

**Affiliations:** ^1^ Research Group of Gastroenterology and Nutrition, Department of Clinical Medicine, UiT the Arctic University of Norway, Tromsø, Norway; ^2^ Department of Gastroenterology, Division of Internal Medicine, University Hospital of North Norway, Tromsø, Norway

**Keywords:** ulcerative colitis, anti-TNF, discontinuation, remission, mucosal TNF

## Abstract

**Background:**

Moderate to severe ulcerative colitis (UC) is generally treated with a step-up algorithm from 5-aminosalicylic acid (5-ASA) to biological agents. There is no general recommendation if or when to de-escalate or discontinue biological therapy. In this study, we performed biological therapy with anti-tumor necrosis factor (TNF) treatment to endoscopic remission followed by discontinuation of therapy. This is a 13- year follow-up study performed for this treatment algorithm.

**Aim:**

This study aimed to assess whether the treatment algorithm outlined above influences the UC phenotype toward a milder form and identify potential biomarkers for altering the disease phenotype.

**Methods:**

Patients with moderate to severe UC were enrolled from 2004 to 2015 and followed up until 2023 to evaluate disease outcomes. Patients were categorized into subgroups based on the highest treatment level required to attain remission: non-biological therapy, biological therapy, or colectomy. Mucosal TNF mRNA expression levels were measured using real-time PCR.

**Results:**

Out of the 116 patients from the original cohort, 71 individuals who had previously undergone anti-TNF treatment to endoscopic remission and subsequently discontinued anti-TNF therapy were included in the present study. Disease outcomes were registered until 2023. By the end of the observation period, 62% of participants were in remission without biological treatment. Among the 71 patients, 39% never experienced a relapse, 23% relapsed but successfully attained remission with untargeted treatment, 18% relapsed and subsequently received a new sequence of biological therapy, and 20% had colectomy. Normalized mucosal TNF mRNA expression was identified as a significant predictor for clinical outcomes.

**Conclusion:**

Most UC patients transitioned to a milder disease phenotype without requiring biological therapy. Treating to normalize mucosal TNF expression emerges as a potential biomarker, predicting the downregulation of disease severity.

## 1 Introduction

Ulcerative colitis (UC) is a chronic inflammatory bowel disease (IBD) with a complex pathogenesis. This involves the interplay of various genes increasing susceptibility to disease development and environmental factors that can induce changes in gene functions (1). The disease severity is commonly categorized as mild, moderate, or severe (2). Treatment for UC typically follows a step-up algorithm, progressing from 5-aminosalicylic acid (5-ASA) via glucocorticoids to biological, targeted agents depending on the success or failure of the steps. The arsenal of highly effective biological agents for treating UC has been expanding, as reviewed by Raine et al. (3) However, there are currently no general recommendations on whether and how to de-escalate biological therapy, neither from the American Gastroenterological Association (AGA) nor the European Crohn’s and Colitis Organisation (ECCO) (3, 4). Consequently, valid criteria for discontinuing biological therapy in UC have yet to be established, emphasizing the need for individualized decision-making (5).

We have previously conducted studies involving IBD patients who discontinued biological therapy. Results suggest that normalization of mucosal tumor necrosis factor (TNF) mRNA expression may serve as a potential biomarker, predicting favorable outcomes following discontinuation of anti-TNF therapy in both UC and Crohn’s disease (6–8).

The present dataset is a further follow-up on a prospective cohort study involving patients treated with anti-TNF therapy. We followed an algorithm with two cardinal deviations from regular biological treatment: first, an intensified induction treatment was applied to obtain endoscopic remission; second, after obtaining endoscopic remission, anti-TNF was discontinued. By extending the observation period beyond the last update in this study from 2019 to 2023 and describing the clinical outcomes, we aim to enhance our understanding of the therapy-modified disease course and potential phenotypic shifts in these UC patients. A secondary objective is to investigate whether our prior findings regarding mucosal TNF mRNA expression remain consistent in this follow-up (9).

## 2 Materials and methods

This is a prospective follow-up of patients who were initially enrolled from 2004 to 2015 in our ongoing transregional study, the Advanced Study of Inflammatory Bowel Disease (ASIB), conducted at the University Hospital of North Norway. The present follow-up spanned from 2019 to September 2023. In essence, all participants in this study were diagnosed with UC according to established clinical guidelines. The patients included in the study were either biologically naïve or had not undergone any other targeted treatment apart from anti-TNF.

The treatment algorithm was a modification of standard infliximab (IFX) treatment: patients underwent the standard induction treatment with IFX 5 mg/kg at weeks 0, 2, and 6. Subsequently, the induction treatment was prolonged giving infusions every 4 weeks until achieving endoscopic remission (Mayo 0–1), instead of the standard every 8-week maintenance regimen, hence referred to as intensified induction. Six patients were subjected to an alternative regimen involving the anti-TNF adalimumab, starting with a dosage of 160 mg, followed by 80 mg weekly until remission, and subsequently, 40 mg every other week. When the patient achieved endoscopic remission, biological therapy was discontinued. Additional criteria of clinical remission > 6 months and normalized mucosal TNF gene expression were included in 2014. In the event of a relapse, retreatment with anti-TNF was administered based on clinical judgment, and the same algorithm was applied.

The severity of the illness was assessed using the Ulcerative Colitis Disease Activity Index (UCDAI/Mayo) scoring system. Colonic mucosal inflammation was graded on a scale of 0 to 3 using the UCDAI/Mayo endoscopic subscore (MES). Moderate to severe UC was defined as a UCDAI score falling within the range of 6–12 (10).

Remission was defined as a reduction in the UCDAI score to less than 3 together with an endoscopic subscore of 0 or 1.

Relapse was defined through either clinical or endoscopic evaluation, or a combination of both. The following criteria were applied: an increase in the UCDAI greater than 3, an endoscopic score greater than 1, or a partial Mayo score exceeding 2, coupled with a calprotectin level surpassing 100. In instances where objective measurements were not available, a relapse was determined based on clinical judgment, prompting therapeutic intervention such as the escalation of medical therapy.

In the present registration of clinical outcomes, patients were categorized as either in remission or experiencing a relapse. Subsequently, the relapse category was divided into subgroups based on the highest treatment levels required to achieve remission: biological therapy, non-biological therapy (including oral and local steroids, as well as 5-ASA and immunomodulators), and colectomy.

Mucosal TNF mRNA expression taken at anti-TNF discontinuation was evaluated as a predictor of outcomes. The methods for measuring and defining normalized mucosal TNF mRNA expression in mucosal biopsies through real-time PCR have been previously described (7). Fe-calprotectin was measured by a kit from Calprest Eurospital, Trieste, Italy (positive test < 50 mg/kg).

### 2.1 Ethical approval

Ethical approval and consent to participate were obtained from all participants, who were informed and who provided written consent The study adhered to the principles outlined in the Helsinki Declaration. The protocol including the establishment of the project biobank, was recommended by the Regional Committee of Medical Ethics of Northern Norway under ref. nos. 14/2004, 1349/2012, and 29895/2020.

### 2.2 Statistics

We conducted Cox regression analysis using IBM SPSS Statistics 24 (IBM Corporation, Armonk, New York, USA) to examine the relationship between the independent variable, mucosal TNF mRNA expression, and the outcome variable, relapse. Initially, the model incorporated covariates such as age, sex, disease distribution, and medication. However, none of these covariates demonstrated a significant impact on the results, leading to their exclusion from the final model.

## 3 Results

### 3.1 Clinical groups and demographics

**Figure 1:**
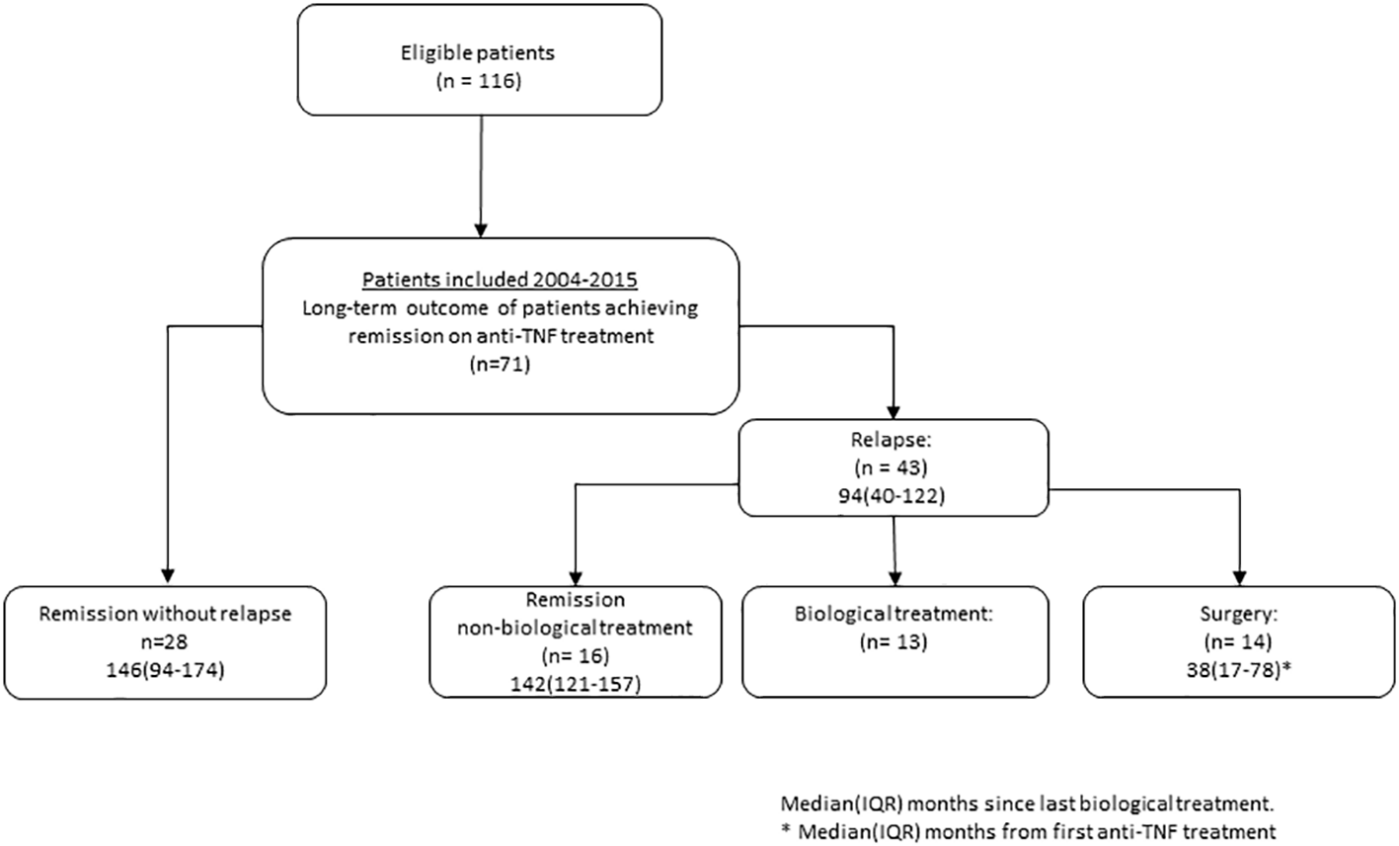
Flowchart of patients with ulcerative colitis treated to remission on anti-tumor necrosis factor (TNF) treatment.

Out of the initial 116 patients recruited for the study spanning from 2004 to 2015, a total of 71 patients were included in the present follow-up. Withdrawals from the study occurred because of reasons such as loss to follow-up, primary non-responsiveness to treatment, and the presence of other diseases. For the patients included, clinical outcomes and demographic characteristics are described in **Figure 1** and **Table 1**.

**Table 1:** Demographic data.

Patient groups	Remission without relapse N=28	Remission non-biological N=16	Biological N=13	Surgery N=14
Age med (IQR)^#^	46 (36–52)	41 (30–56)	43 (33–48)	38 (23–47)
Sex: female/male	14/14	3/13	6/7	5/9
Colonic area P/L/E	6/12/10	2/7/7	0/7/6	2/3/9
Calprotectin at anti-TNF discontinuation med (IQR)	27 (20–80)	45 (26–108)	65 (21–127)	132 (32–230)
Calprotectin in follow-up med (IQR)	54 (25–96)	34 (20–114)		
Mucosal TNF copies/μg mRNA at anti-TNF discontinuation med (IQR)	9500 (6000–16800)	11400 (6200–17800)	9200 (3900–16900)	16300 (9600–26500)
Medication		*		
5-ASA	27	16	11	
IMiDs	12	2	5	

med (IQR), median (interquartile range 25–75) and P/L/E, proctitis/left-sided/extensive.

IMiDs, immunomodulatory imide drugs.

#Age at anti-TNF discontinuation.

*Seven patients received short courses of prednisolone; two patients received budesonide enema; and seven patients adjusted the dose of 5-ASA or additional 5-ASA suppositories or enema.

### 3.2 Long-term remission

**Figure 2:**
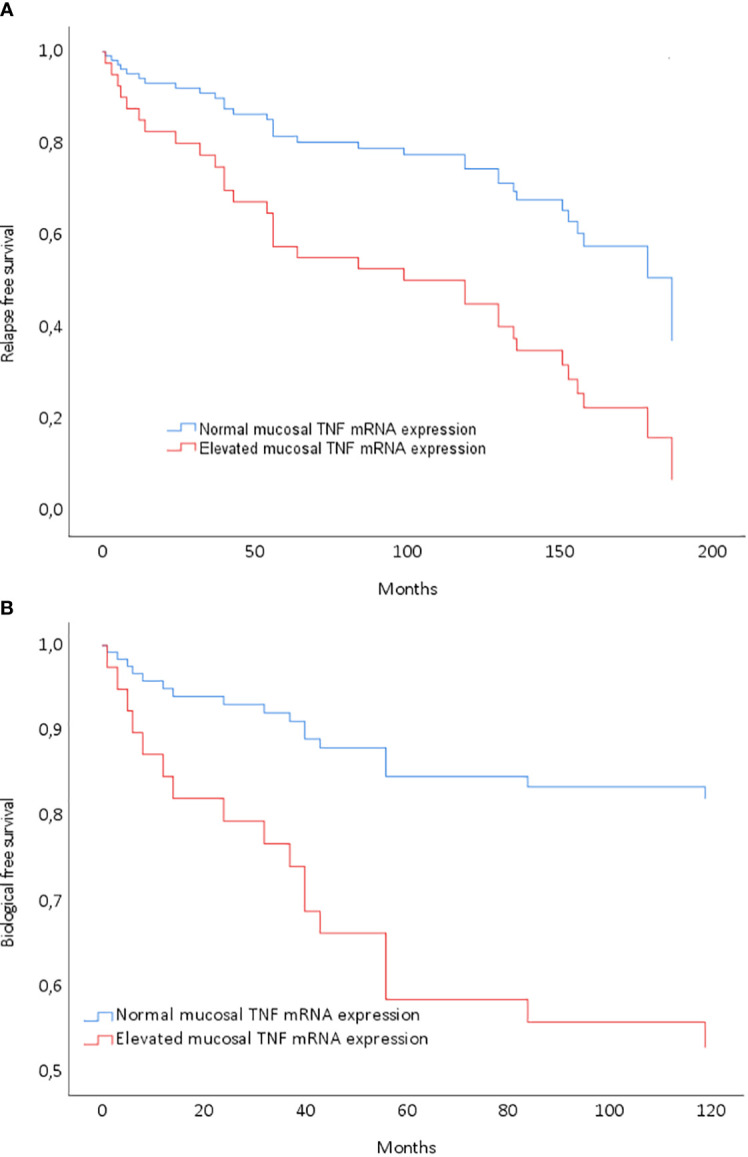
Cox regression model entering mucosal tumor necrosis factor (TNF) gene expression normalization after the last anti-TNF treatment. **(A)** Model *P*=0.02; low TNF at infliximab (IFX) discontinuation: HR 2.71 (1.25–5.88) for relapse-free survival (*P*=0.01). **(B)** Model *P*=0.02; low TNF at IFX discontinuation: HR 3.22 (1.06–9.80) for biological therapy-free survival (*P*=0.04).

Long-term remission without relapse was noted in 39% of the patients (28/71), with a median observation time of 146 months (range: 94–174) after discontinuation of anti-TNF. All patients in this group were using 5-ASA, and six of them were also on immunomodulatory imide drugs (IMiDs). Notably, mucosal TNF mRNA expression emerged as a significant predictor of achieving long-term remission without relapse following anti-TNF discontinuation (see **Figure 2A**).

### 3.3 Biological therapy-free remission

Biological therapy-free remission was achieved by 23% of the patients (16/71), with a median observation time of 142 months since discontinuing biological treatment. All individuals in this subset were using 5-ASA, and three were additionally on IMiDs. They had received short courses of prednisolone for flares to induce remission, as outlined in **Table 1**. By the end of the observation period, 62% of the patients (44/71) were in remission, encompassing both long-term remission and biological therapy-free remission. Importantly, mucosal TNF mRNA expression emerged as a significant predictor of achieving remission without the necessity for further biological treatment (**Figure 2B**).

### 3.4 Biological treatment

Relapse with a need for anti-TNF or other biological treatment was observed in 18% of the patients (13/71), without the need for surgery.

### 3.5 Surgery

Colectomy was performed in 20% of the patients (14/71) with a median observation time of 38 months (range 17–78) since first induction treatment.

## 4 Discussion

We present a 13-year prospective follow-up study of moderate to severe UC, employing an algorithm of intensified anti-TNF treatment until remission was achieved, followed by discontinuation of biological therapy. Throughout the observation period, 62% of the patients did not require biological re-treatment, including 39% who exhibited no signs of relapse and 23% who experienced relapse but achieved remission through the escalation of untargeted treatment. The remaining patients either received additional biological therapy (18%) or underwent colectomy (20%). The results indicate that anti-TNF treatment may induce a shift in the disease phenotype toward a milder form, often rendering 5-ASA as the primary treatment for many patients. This study contributes to existing literature demonstrating that the normalization of mucosal TNF mRNA expression can serve as a potential biomarker and a therapeutic target in a treatment algorithm involving the discontinuation of biological therapy.

It is generally accepted that the most suitable treatment target is achieving complete mucosal healing (3, 11). It is well-known that endoscopic inflammation often persists beyond clinical remission (12, 13). Over the last two decades, the definition of remission in UC has evolved. Terms like mucosal healing have undergone redefinition. Initially defined as a Mayo endoscopic subscore (MES) ≤ 1, mucosal healing is more recently characterized as a MES of 0, reflecting a shift toward stricter therapeutic targets with emerging evidence supporting more favorable outcomes (14, 15). Mucosal healing as a criterion for the discontinuation of biological therapy has shown high rates of relapse, ranging from 23% to 52% (16–18). In a large observational study discontinuing anti-TNF treatment in UC with clinical remission, the rate of relapse was 19% per patient year (19). Nevertheless, to the best of our knowledge, no randomized controlled trial (RCT) has been undertaken to directly compare the discontinuation versus maintenance of biological therapy in UC. Consequently, the suggestion for discontinuing biological therapy in UC is best considered after achieving a prolonged period of stable disease remission (5).

Histological remission has been proposed as a potential therapeutic target, with emerging evidence suggesting improved clinical outcomes (20). Despite this, there is currently a lack of studies focusing on histological remission as a criterion for discontinuing biological therapy. Recently, a novel definition of remission, termed “disease clearance,” has been introduced in a study. This comprehensive approach combines endoscopic, clinical, and histological remission. The study demonstrated enhanced outcomes, with a lower risk of hospitalization and surgery observed in patients with UC who achieved disease clearance. The study also highlighted the impact of achieving early disease clearance (21). An intriguing consideration is the potential efficacy of a top-down strategy in biological therapy for UC compared to a step-up approach, although solid documentation supporting this assertion is currently lacking. Our approach of intensified biological treatment leading to remission may serve as a step toward implementing a top-down strategy. Regardless of the chosen algorithm, the overarching objective of achieving a swift induction of remission is likely the optimal path for attaining long-term remission and preventing pharmacodynamic refractory mechanisms (22). It is crucial to note that while this hypothesis is promising, it remains to be documented and requires further investigation in future studies. Furthermore, an intriguing aspect to explore would be whether achieving histological remission upon discontinuation of anti-TNF therapy could retrospectively serve as a predictor for long-term outcomes. This specific inquiry is currently underway within our research group.

UC patients in long-term remission with a MES of 0 or 1 still express increased mucosal pro-inflammatory cytokines and reduced anti-inflammatory cytokines compared to healthy controls (23). Despite the promising results of our treatment algorithm with endoscopic remission, our patients are most likely far from being in immunological remission. A prior analysis conducted on a highly selected group of patients, previously treated with anti-TNF and in very prolonged remission, revealed that various mucosal gene transcripts, encompassing both pro- and anti-inflammatory markers, did not normalize when compared to healthy controls (9). This finding may provide insights into why some UC patients experience relapses necessitating surgery.

The central inquiry revolves around the comparative effectiveness of our treatment algorithm versus maintenance therapy. In a maintenance study involving primary responders to anti-TNF, both adalimumab and infliximab exhibited a secondary loss of response of 60% over 139 weeks and 159 weeks, respectively (24). Another study reported a relapse rate of 61% among UC patients undergoing 5 years of infliximab maintenance therapy (25). Consequently, the encouraging outcomes from our treatment algorithm suggest a greater efficacy in preventing new disease relapses.

The colectomy rate in our study, after achieving remission was 20% after 12 years. There are limited long-term observational studies on colectomy rates in UC patients treated with anti-TNF. In a 5-year observational study conducted by Arias, the colectomy rate was 20%, while in another study, the colectomy rate reached 40% after 36 months (25, 26). The evidence from our treatment algorithm suggests a favorable trend toward reducing the risk of colectomy.

Within our research group, we have gathered compelling evidence demonstrating the impact of mucosal TNF expression on clinical outcomes, particularly when discontinuing biological therapy in both Crohn's disease (CD) and UC (6–9). The present data align with earlier observations in the longitudinal study spanning from 2004 to 2019, where the cohort experiencing long-term remission exhibited the lowest TNF transcript levels. However, it is noteworthy that these levels remained above the normal range in many patients. This underscores the importance of considering the normalization of mucosal TNF transcripts as a pivotal criterion in the decision to discontinue biological therapy.

Our treatment algorithm indicates that obtaining endoscopic remission in UC changes and downregulates the phenotype toward a milder disease although some of the patients were still on immunosuppressive treatment. This could mean improved mental and physical health and quality of life for the patients, especially in social functions. Ultimately, this results in reduced disease burden and decreased costs, both for the patients and the healthcare system.

The strength of this study lies in its extended observational period, a substantial cohort of carefully selected patients with moderate to severe UC treated with biological therapy to endoscopic remission, and the minimal loss to follow-up registrations. One limitation is that control endoscopy was only conducted when clinical and fecal calprotectin data were uncertain. The most apparent weakness is the absence of an RCT design. Notably, the treatment algorithm involving discontinuation, coupled with the normalization of mucosal TNF transcripts as a criterion, is an ongoing prospective study initiated in 2015. The potential inclusion of histological remission as a criterion for enrollment is also under consideration.

## 5 Conclusion

The treatment algorithm used in moderate to severe UC shifts the disease phenotype to a milder form, without requiring retreatment with biological therapy in over 60% of the patients. Moreover, the long-term risk of both flares and risk of colectomy are low compared to maintenance therapy. There is a compelling need for an RCT discontinuation study design, wherein the normalization of mucosal TNF transcripts should be one of the several inclusion criteria.

## Data availability statement

The raw data are available on shared Mendeley Data folder on https://data.mendeley.com/datasets/sfzz7dbshj/1.

## Ethics statement

The studies involving humans were approved by Regional Committee of Medical Ethics of Northern Norway Ref No: 1349/2012. 29895/2020. The studies were conducted in accordance with the local legislation and institutional requirements. The participants provided their written informed consent to participate in this study.

## Author contributions

JF: Conceptualization, Writing – original draft, Writing – review & editing. RG: Conceptualization, Writing – original draft, Writing – review & editing. KJ: Conceptualization, Formal analysis, Supervision, Writing – original draft, Writing – review & editing.
